# Supplementary material for: Diversity impact on organizational performance: Moderating and mediating role of diversity beliefs and leadership expertise
Source: PLoS One. 2022 Jul 25;17(7):e0270813. doi: 10.1371/journal.pone.0270813 (PMC9578724; doi:10.1371/journal.pone.0270813)
Supplement: Attachment — Submitted filename: PLOS Review_2021.docx [file pone.0270813.s001.docx]

The study finds out the impact of diversity in reference to variables like gender, age, educational background, and ethnicity on organizational performance. It also investigates the moderating role of diversity beliefs and mediating role of leadership expertise was assessed on Organizational performance. The study is interesting, and the results are useful for diversity related research. However, the manuscript needs to be thoroughly checked for language including grammar and presentation. Please find below my specific comments:

1. The in-text citation needs to be corrected at several places.

2. The first paragraph of the introduction needs addition of some citations. For example, adding a reference to the statement- “Workforce diversity has been identified as one of the strategic capabilities that add value to the organizations over their competition”

3. Rephrasing the objective in the abstract section in accordance with what has been stated in the introduction section will make the objective sound clearer. The objective stated in the introduction section says: “Keeping in view the gaps and recommendations, the main query of the research is that “whether leadership expertise adds to the organizational performance?” and what value diversity beliefs can add to organizational performance? Therefore, our contribution to the diversity literature is threefold. First, we attempt to better understand and assess the impact of diversity on organizational performance. Second, this study has leadership expertise as a mediating variable. Thirdly, in order to determine the extent to which diversity and organizational performance are related, the diversity beliefs are used as a moderating variable on the recommendations of previous researchers”. The abstract must clearly reflect that the study has threefold objective.

4. Adding a small paragraph on how the statistical method (path estimation) used for this study is a best fit. This would provide a strong support to the result’s credibility.

5. The figures could be improved in terms of size and quality.

6. Not all the references cited in the text are in the reference list. Make sure to include only those articles that are relevant and are used for making reference for your ideas.
