# Supplementary material for: Diversity impact on organizational performance: Moderating and mediating role of diversity beliefs and leadership expertise
Source: PLoS One. 2022 Jul 25;17(7):e0270813. doi: 10.1371/journal.pone.0270813 (PMC9578724; doi:10.1371/journal.pone.0270813)
Supplement: Attachment — Submitted filename: PS Paper-01.docx [file pone.0270813.s002.docx]

**Diversity Impact on Organizational performance: Moderating and Mediating role of Diversity Beliefs and Leadership Expertise**

Dr. Jamshid Ali Turi, Assistant Professor, Department of Management Studies, Bahria Business School, Bahria University, Islamabad, Pakistan

Email: [Jamshidali.buic@bahria.edu.pk](mailto:Jamshidali.buic@bahria.edu.pk)

Sudhaishna khastoori, Department of Management Sciences, SZABIST Larkan, Pakistan

Email: [sudhaishna.khastoori@gmail.com](mailto:sudhaishna.khastoori@gmail.com)

Dr. Shahryar Sorooshian, Associate Professor, Department of Business Administration, University of Gothenburg, Sweden

Email: [shahryar.sorooshian@gu.se](mailto:shahryar.sorooshian@gu.se)

Corresponding Author:

Dr. Shahryar Sorooshian, Associate Professor, Department of Business Administration, University of Gothenburg, Sweden

Email: [shahryar.sorooshian@gu.se](mailto:shahryar.sorooshian@gu.se)

**Diversity Impact on Organizational performance: Moderating and Mediating role of Diversity Beliefs and Leadership Expertise**

**Abstract**

Diversity at workplace has been recognized as one of the strategic competencies that would add great value to organizations performance and give competitive advantage. As Pakistan is globally growing country, it attracts extremely innovative and highly skilled people to work here. Gender, age, educational background, and ethnicity are considered as most commonly diversified demographic variables seen among the employees of many organizations. Therefore, the current research pays attention on the impact of diversity with reference to these four independent variables. If diversity is managed properly, it would generate constructive results and vice versa. In the paper, moderating role of diversity beliefs and mediating role of leadership expertise was assessed on Organizational performance. Quantitative method was used to comprehend the objectives of research. A self-administered questionnaire, using 7-likert scale approach was adopted to collect the responses from 176 employees. Results signpost impact of gender, age, educational background, and ethnicity on organizational performance. SPSS and SMART-PLS3 was used for data analysis. The findings indicate that only diversity has statistically significant impact on the organizational performance. Moreover, moderating variable diversity belief has no effect on organizational performance, leadership expertise plays significant mediating role in organizational performance.

***Keywords:*** *Organizational performance; workplace diversity; leadership expertise; diversity beliefs; age diversity; gender diversity; educational background diversity; ethnicity*

**Introduction**

Internet and Globalization have minimized the gap in the market scale between large Multi-National Corporations and Small and Medium Enterprises. Among the major challenges in the world, managing the workforce diversity has become a major challenge for management, especially in the organizational context. The today’s workforce has been completely changed in terms of composition and diversity has become the most important subject for organizations competing nationally and globally. Workforce diversity has been identified as one of the strategic capabilities that add value to the organizations over their competition.

In modern organizations, diversity is observable in human aspects, such as, religious, language, age, gender, ethnicity, education, cultural and personality orientation (Ng & Sears, 2020; John F. et al., 2017). The concept of diversity in organizations has changed more to strategic targets. A number of current leaders from large organizations believe that diversity has a great effect on overall organizational performance (Joubert, 2017). Organizations are promoting to hire workers from minorities having different social and cultural backgrounds and also of different gender to improve organizational performance (Hennekam, Bacouel-Jentjens, & Yang, 2019). However, in many cases the managers are not equipped well to practice and manage the diversity effectively, or to examine what factors / components play important role towards valuable diversity management and tasks that can deal with diversity-related issue within workplace (Dahanayake, Rajendran, Selvarajah, & Ballantyne, 2018). Many studies recommend, that diversity exist in different forms with different intensities, and at workplace has potential to harm morale, intensify turnover and may result in substantial communication problems. Therefore, it need to be explored with different factors, in different settings to get its applications and managerial implications for sustainable organizational performance (Tamunomiebi & John-Eke, 2020; Park, 2020; Mousa, Massoud, & Ayoubi, 2020). {Add sentence}

Due to lack of diversity training and understanding diversity beliefs, especially in the developing countries, having rigid social and cultural bounds, leads to organizational biasness. As per (Sahar E-Vahdati, Norhayah Zulkifli & Zarina Zakaria, 2018), recommendations, firms to throw emphasis on corporate governance, accountability, ethics, trust and diversity, due to the fact that problems associated with diversity have yet to be solved. Moreover, organizations also need diversity for rational decision making and promoting conducive environment, where everyone’s beliefs will be given respect, then ultimately employees will self-reflect all the positive benefits (Urbancová, Hudáková, & Fajčíková, 2020; Roberson, 2019). On the contrary, if workforce diversity is mismanaged; this could lead to formation of diverse groups, which may result in emotional conflicts, miscommunication, and power struggle and eventually to higher employee turnover, then diversity workforce would become an inhibitor for organizational development (Bizri, 2018; Jin, Lee, & Lee, 2017).

Given these mixed results additional research on diversity is needed for its further comprehension and explanation. Moreover, previous researches have considered various diversity dimension to identify its impact on organizational performance, however, these studies have not been conducted in projectized environment and have considered role of leadership expertise and diversity beliefs. A previous research was conducted to find out the relationship between Top Management Team functional diversity and firm’s performance with the moderating role of top management (C.E.O) attributes (AnaGarcía-Granero, Fernández-Mesa, Jansen, & Vega-Jurado, 2018; Georgakakis, Greve, & Ruigrok, 2017). It was recommended in this study that upcoming researches can be conducted on leadership’s style of managing diversity inside the organizations. Keeping in view the gaps and recommendations, the main query of the research is that “whether leadership expertise adds to the organizational performance?” and what value diversity beliefs can add to organizational performance? Therefore, our contribution to the diversity literature is threefold. First, we attempt to better understand and assess the impact of diversity on organizational performance. Second, this study has leadership expertise as a mediating variable. Thirdly, in order to determine the extent to which diversity and organizational performance are related, the diversity beliefs are used as a moderating variable on the recommendations of previous researchers.

Furthermore, while doing in-depth analysis on this research topic, the previous studies have understood diversity more in terms of discrimination and racial prejudice; however, these terms give negative description. In today’s age of globalization leading competition, diversity is much more than race and class differences and if harnessed effectively, it can result in huge productivity and organizational success. In addition to this, the diversity management has not gained much attention practically while addressing diversity workplace and I believe that diversity management is more important in order to manage the diverse workforce to gain desirable organizational results.

**Literature Review**

Diversity is explained as considering; recognizing & giving respect to others opinion & differences irrespective of their culture, gender, age, social status, race, physical capability & so on (Heavey & Simsek, 2017; Bizri, 2018). Every individual is unique in their own ways, and one may not find exactly two individuals alike in the whole universe (Ali & M.Konrad, 2017). There can be many aspects of diversity, but core differences among individuals are gender, age, education, ethnicity and religion. Diversity is these days considered as a strength of any organizations as compared to previous decades. It is used as a force to dig opportunities, face challenges and to explore new avenues and directions (ZhenShao, Feng, & Hu, 2017). Moreover, diverse background enhances knowledge and skill level, help to understand behavior, conflicts and filled the gaps (Gomez & Bernet, 2019; Bizri, 2018).

In the today’s era where many organizations have incorporated diversity (Dahanayake, Rajendran, Selvarajah, & Ballantyne, 2018). The concept of diversity in organizations has changed more to strategic targets. A number of current leaders as of large organizations believe that diversity has a great effect on overall organizational performance (Wang & Dass, 2017; Gomez & Bernet, 2019). Businesses and the projectized organizations need to adopt a diverse workforce for understanding the needs of rapidly growing minority consumers more effectively (Campbell Leaper, 2011). According to Bizri (2018) and Heavey & Simsek (2017) diversity beliefs refer to a person’s perception or preference towards a homogenous group or heterogeneous group and person’s diversity beliefs may possibly be one of the factors influencing between organizational performance. This concept supports that diversity has a positive effect on Organizational performance when supported by positive beliefs and values (Manoharan & Singal, 2017).

Advancement in technology has brought the people closer from all around the world than ever before. Due to these educational institutes, businesses and other organizations are looking for better ways to serve their consumers and stakeholders (Nuscheler, Engelen, & Zahra, 2019). Diversity is believed to be the source of competitive advantage through creating a better organizational image, enhancing organizational performance, attracting and retaining the qualified and multitasking employees. Investing the workforce diversity in organizations is carried out throughout the world (Kalargyrou & Costen, 2017; Bizri, 2018).

According to recent study carried out by Kundu & Mor (2017), on sample size of 278 managers chosen from leisure industry, his findings concluded that the general positive view of workforce diversity can result in positive impact on organizational and new venture (project) performance. In another research of 391 managers taken from 130 firms revealed that the perception of employees about workforce diversity is positively linked with the organizational performance (Duchek, Raetze, & Scheuch, 2020; Ou, Seo, Choi, & Hom, 2016). Moreover, in another study conducted by Pitts (2009), he found that employees perceived their organization more effective when they were positively responsive to diversity management (Gomez & Bernet, 2019). But diversity refer different in different organizational and cultural setting, therefore, renders different results and perspective, and need to be managed in a special way (Jankelová, Joniaková, Procházková, & Blštáková, 2020; Dahanayake, Rajendran, Selvarajah, & Ballantyne, 2018).

**Gender Diversity**

Gender diversity refers to the emotional difference and experience that is publicly and culturally attached to being men and women within any firm (Zhao, Chen, Jiang, & Shi, 2017). The success of any organization depends upon fair practices followed within organizations, and gender equality and equity is one of them, implying that men and women should be treated and promoted equally in the workplace (Zahid, et al., 2020; Mun & Jung, 2018). Despite of the fact, the world is transforming since decades, but we are way behind achieving the true gender differences and equality within organizations (Zahid, et al., 2020). The gender oriented inequities within workplace are reinforced by the personal biases and stereotypes, referring that the status of a men is perceived higher than women. Many organizations prefer hiring male employees because they perceive men to be the better performers (Luanglath, Ali, & Mohannak, 2019).

According to McKinsey Report, 40% of the women are employed in workforce globally and only 5% of them are on the top positions, surprisingly women earn 80% as compare to their male colleagues, which means that if a male accountant has served any organization for 5 years and is getting salary of 70,000/- and women in the same organization on the same position with equal five years’ service gets 56,000/- and these statistics indicates clearly the impact of gender diversity at workforce (Kan & Kaplan, 2019; Kato & Kodama, 2018 ; Krivkovich, et al., 2018). According to the Kato & Kodama (2018 ), if the gender issues within organizations is not recognized and managed during preliminary phases a significant number of workforce diversity will remain ineffective. The research also states that the most difficult challenge is to deal with the perception of women not being equal to the men. According to United Nations Report, in 2015 only 50% of women were working worldwide as compared to the 77% of men population (Zahid, et al., 2020). However, providing equal job opportunities to women is highly important for enhancing performance of workers in an organization. Organizations that failed to execute the fair employment practices faced higher costs (Madsen, Townsend, & Scribner, 2020; Toscano, Giusino, & Pordanjani, 2002; Bizri, 2018).

**Age Diversity**

Age diversity talks about the capability of any organization for accepting different age groups. Business environment can only grow and succeed when it has the ability to accept various age groups within an organization and adjust age diversity in different ways (Profili, Innocenti, & Sammarra, 2017; Seong & Hong, 2018; Campbell Leaper, 2011). Since many decades’ workforce was facing age diversity issues but now it has gained significant consideration because professionals are ready to work till their late 60’s and youngsters want to start their work life in their early ages, and they even work willingly as a part timers with their on-going studies (Oliveira & Cabral-Cardoso, 2017; Wang & Fang, 2020; Veronica, Alamsjah, Elidjen, & Kosasih, 2020). Organizations are welcoming this trend because they need both, experienced and trained employees as well as young talent with innovative mind-set, for better organizational performance, new ventures singings and Organizational performance (Sakdiyakorn & Wattanacharoensil, 2018; Barros, 2019).

**Ethnic Diversity**

Ethnic diversity refers to difference in religion, language and cultural background of employees in any organization (Kundu & Mor, 2017). In today’s world, organizations are focusing on building diversity to make stronger organizational adaptableness, increase competitive advantage and decrease legal risks. In spite of this, many companies are still in struggle of making policies and to reduce negative impact over organization’s performance due to ethnic discrimination (Khan, et al., 2019; Hsiao, Ma, Lloyd, & Reid, 2019). According to EEOC data collection, $112.7 million were collected annually from employers due to ethnic diversity violations (Hennekam, Bacouel-Jentjens, & Yang, 2019). Contrarily, ethnic diversity can be in favor of organization as well, such as, employees from different backgrounds when work in the same organization represent different lifestyles, experience various cultures, beliefs and skills that organization utilize in making strategic decisions more effectively (Dahanayake, Rajendran, Selvarajah, & Ballantyne, 2018). Due to these attributes, organizations have multiplicity of diverse people and ultimately it favors the organization’s performance (Jansson & Bursell, 2018; Laurence, Schmid, & Hewstone, 2018).

**Educational Diversity**

Educational diversity refers to differences knowledge, training, skills, experience & qualification, an individual carry (Lindsay, Rezai, Shen, & Lyons, 2020; Gomez & Bernet, 2019). New business organizations don’t want to hire all the qualified personal, for better and smoother performance, they mix soft and hard-tech personal to deliver best in the challenging situations (Madsen, Townsend, & Scribner, 2020). Normally, it is considered that, organizations with less education, skill, experiences and training performance remain slower as compared to organization with highly qualified personals (Luanglath, Ali, & Mohannak, 2019). Many times, organizations refuse to employee workers with high qualifications, because people with higher education’s not always perform better as compared to low education attainders’. Although educational diversity is significant to get a desired job (Martinez, Zouaghi, & Marco, 2017; Makudza, Muchongwe, & Dangaiso, 2020).

**Organizational Performance**

Major aspects of the organizational performance are timely completion of the project and operations within the scope, quality and budget (Albert, Balve, & Spang, 2017; PráMartensa, Machado, Martens, Silva, & Freitas, 2018). Moreover, organizations set certain standards for their assignments, jobs and operations, and try to get their results and even operations within the defined limits to meet organizational key performance indicators (Barros, 2019). But many researchers have raised voices that, if the organizations should focus on their internal and external stakeholders’ expectations, needs and requirements, otherwise, their performance may not be considered complete, perfect and successful (M.Carvalho & Jr, 2017). Moreover, adherence to the government agencies and other regulatory authorities’ compliance is also needed for the successful implementation of the project and their deliverables for all organizations, irrespective of their domains, size and context (Butticè, Colombo, & Wright, 2017). Diversity in organizations is really appreciated around the globe, because it overcome the complex situations, help in finding better solution to different arising challenges. Diversity bring diversity in skills, which premised better organizational performance and timely completion of different assignments (Radujković & Sjekavica, 2017). But, at the same time, content analysis also proclaims that diversity create role and identity conflicts among organizational workers, which worsen organizational performance and question its timely completion of different operations and projects (Wua, Liu, Zhao, & Zuo, 2017). In different area around the globe, large, complex and even small organizations are having diverse experiences, results, outputs and outcomes due to diversification in the knowledge workers. Therefore, there is no one formula for handling diversity in the project and organizational context, but every organization may need special techniques, to manage and handle diversity (Páez-Avilés, Rijnsoever, Juanola-Feliu, & Samitier, 2018; PrósperoSanchez, Terlizzi, & Moraes, 2017).

**Diversity beliefs**

Diversity beliefs refer to different ways of doing things with reference to one’s own values and principles (Makudza, Muchongwe, & Dangaiso, 2020). Diversity beliefs refers that different people across the world believe different things, practice different religions and follow one’s own cultural attributes (Mousa, Massoud, & Ayoubi, 2020). Today, globalization in one of driving forces of diversity within organizations. However, the accommodation of diversity beliefs in terms of spiritual, cultural, and political views sometimes poses a challenge in a diverse organization (Profili, Innocenti, & Sammarra, 2017; Park, 2020). Personnel need to be reminded that they should not impose their opinions on others in order to prevent disputes and spats. Also, they need to ensure their personal and ethnic beliefs independent of their work obligations (Tsui et al., 1992; Oliveira & Cabral-Cardoso, 2017). The employment practices linked with unbiased diversity beliefs can lead to constructive organizational results (Mousa, Massoud, & Ayoubi, 2020; Seong & Hong, 2018).

**Leadership expertise**

The proficiency of a leader is to lead a team in right direction irrespective of diverse organizational culture to achieve organizational objectives and targets (Sinnema, Daly, Liou, & Rodway, 2020). Leadership is supposed to give new directions, new philosophies, hope, optimisms and enthusiasm to the knowledge workers. Therefore, leadership is expected to play important role in every organizational performance by knowing their staff and taking care of them like a man. Leadership may slaughter whole workforce, decay their moral, capacities and capabilities, if that is not supported with latest human and technological skills. Leadership expertise include, but not limited to 1). Specific subject matter details, 2). Broad and deep knowledge of the field, 3). Knowledge of the outside world and 4). Political skills and understandings. Moreover, to manage the changes, leaders are supposed to response according to the market demands and stakeholder’s expectations. A prosperous leader should know what type of leadership is expected and implement it consequently (Kalargyrou & Costen, 2017). People with diverse backgrounds may differ in their expectations and conception of leadership. In today’s era of competition, a sustainable benefit of globalization relies on abilities and skills of a leader who can deal with diversity and execute progressively complicated business strategies. The efficient organizational diversity management is a central to Organizational performance (Jeong, 2020; Seo, Huang, & Han, 2017).

After brief introduction, the Table 1 represent a summary of the studies conducted on diversity in the chronological order.

Table 1

Previous studies on Diversity

| **Sr: #** | **Title** | **Summary of Findings** | **Researchers** |
| --- | --- | --- | --- |
| 01 | Global ageing – meeting the business challenges | Organizations are capable enough to utilize the capabilities of older employees, owing to wrong stereotypes that they are prone to health issues, cannot adapt to change and generate poor outcomes than younger employees. | (O’Reilly, C.A., Williams, K.Y, 1998) |
| 02 | Group democracy and innovation: Does diversity help? | There is no distinct relationship between Organizational performance and age diversity. This study was consistent with the results of former researchers, claiming that a heterogamous age group is more dynamic than homogenous group. | (Richard, O. C., 2000)  (Michael A. Silverstein, 2008) |
| 03 | Gender diversity, ethnic diversity and employee performance in a South African higher education institution | According to findings, ethnic and gender diversity both have significant positive relationship with employee performance, which eventually improves organizational performance. | (Jackson et al, 2003) |
| 04 | Recent Research on Team and Organizational Diversity: SWOT Analysis and Implications | On the contrary, another research examined that teams with greater ethnic diversity generates poor performance in comparison to teams with similar beliefs and values. | (Ilmarinen, J., 2005)  Klein, (2005). |
| 05 | Workforce Diversity and organizational Performance | More gender diversity among employees brings a spectrum of skills, perspectives, and knowledge to the firms that can tap into and base firms’ strategic goals up. | (Knippenberg et. al, 2008) |
| 06 | What's the Difference? Diversity Constructs as Separation, Variety, or Disparity in Organizations | This study suggested that moderate level of ethnic diversity is positively associated with innovation, while greater level of ethnic diversity might have negative impact since it can give rise to cliques and conflict attributable to social categorization. | (Rolf, V. et al, 2008) |
| 07 | The Diversity – Validity Dilemma: Beyond Selection – the role of affirmative action | The employers mainly do not employ those whose education is insufficient. Therefore, the organization performance declines. Employee’s having strong education base are more imaginative and able to accomplish organizational goals and objectives. | Tracy and David, (2011) |
| 08 | The impact of workforce diversity towards employee performance: Evidence from banking sector of Pakistan | According to former researches, for employees the educational background is significant to get a desired job, without educational background employees can’t find a satisfactory job. | (Rizwan, 2016). |
| 09 | Group diversity and group identification: The moderating role of diversity beliefs. | Former researches have argued that similar diversity beliefs encourage respect, acceptance, and teamwork regardless of the differences in age, education, gender, religion, native language or communication styles of workforce. Whereas, dissimilar diversity beliefs raise conflicts and hinder the organizational performance. | (Okoro, 2012) |
| 10 | Diversity and Its Impact on Organizational Performance: The Influence of Diversity Constructions on Expectations and outcomes | A sustainable benefit of globalization relies on abilities of a leader who can deal with diversity and execute progressively complicated business strategies | (Ali, et al., 2013) |
| 11 | Support of workplace diversity policies: The role of race, gender, and beliefs about inequality | Employees having varied beliefs may perceive value in the idea of working with diverse people who are different from one another is favorable for organizations. | (Gupta, R., 2013) |
| 12 | Racial diversity, business strategy and firm performance: A resource based view. | The importance of organizational leadership together with the realization strategy constitutes the basis of competitive advantage. | (Michael A. Silverstein, 2008) |
| 13 | Group information elaboration and group decision making: The role of shared task representations | This study supports that employees and management sharing heterogeneous beliefs induces positive behavior and add various traits that increases the organizational performance. | (Seong, J. Y, 2015) |
| 14 | Workforce diversity and firm performance: Relational  coordination as a mediator and structural empowerment and multisource feedback as moderators | This research affirmed that education develops knowledge, skill, and ability (KSA) based perceptions – a complementary fit. | (Simbarashe Zhuwao, Hlanganipai Ngirande, Wiseman Ndlovu , and Sam T. Setati, 2019) |
| 15 | Workforce diversity organizational performance. | In a research, it was examined that moderate level of gender diversity boosts the competitive edge, whereas greater level of gender diversity reduces the organizational performance. | (William at al., 2019) |

**Theoretical Framework and Research Hypothesis**

**I.	Social identity theory**

This theory was introduced by John Turner and associates (Hogg & Terry, 2000). These theories describe how to manage diversity in workplace at micro and macro level for rendering better performance inside the organization and in different projects (Bizri, 2018; Kato & Kodama, 2018 ). Study findings indicates that social identity create identification, motivations, task performance and context, which leaves positive impacts on Organizational performance (Jansson & Bursell, 2018; M.Carvalho & Jr, 2017). Beside social, it also helps in cognitive development, which polish workers’ skill, knowledge and experiences, which are pre-requisites for boosting performance in the projectized organization (Jin, Lee, & Lee, 2017; Ou, Seo, Choi, & Hom, 2016).

Social identity theory state the behaviors of the individual in isolation as well as in a group. It explains how the worker in the organization specify role for their selves, based on their preferences, interests, like and dislikes. It explains the behaviors, group and also of the organization (Trepte & Loy, 2017). This theory can be further expended to self-categorization, social identification and social comparison. It is natural phenomenon, that worker inside the organization, place themselves in different categories, based on their likes, interest, skills, qualifications, experiences, social, cultural, age, religion etc. This process is called self-categorization. self-categorization process give birth to self-esteem, social identity, belongingness, social influence and social identification, which prevails better results on organizational performance. Moreover, it also develops opportunities for competition among different stratus among the project and organization, it helps in attaining timely targets with in a scope and budgets (Hornsey, 2008; Campbell Leaper, 2011). However, this social comparison may also lead to role conflicts or role avoidance, which may create hurdles in smoother project and operations execution and implementation. At the micro level or individual employee level, is based on managing and understanding stereotypes, discrimination, and prejudices (Hogg, 2001). The supporters of managing the diversity in organizations at micro level also feel that stereotypes and prejudices are immediate predecessors for discrimination in organizational performance (Joubert, 2017). Moreover, discrimination is either controlled through controlling stereotypes and prejudices or eliminated completely. According to Kan & Kaplan (2019) it is also argued that there are some other precursors of discrimination, which includes I) Interact with demographically different people, II) Experience of realistic group conflict, and III) The development of social categorizations and social identities. Controlled and managed diversity at micro level rendered better results due to their proximity, easily managed and controlled operations, responsibility, accountability, role identity and risk avoidances (Ortiz & Redmon, 2020; Yasmeen, Ahmad, Raziq, & Laeeq, 2020; AndreSøraa, et al., 2020; Zaman, 2020).

In contrast, at the macro level or managerial level of business, the objective of managing diversity is to observe and moderate the relationship between Organizational performance and workplace diversity. Nonetheless, there are few models at macro level for managing the diversity, where the model formulated by Thomas and Ely (1996), is used extensively (M.Carvalho & Jr, 2017). The model of Thomas and Ely emphases on the relationship, which diversity at workplace has on outcomes of the business (Profili, Innocenti, & Sammarra, 2017). In order to manage diversity, Thomas and Ely introduced three types of views at organizational level: access - and - legitimacy, fairness - and – discrimination, and integration - and - learning (Nuscheler, Engelen, & Zahra, 2019). Among these three perspectives, they found that teams perform best under the integration - and - learning process. This perspective focuses on creating positive relations within groups, sense of being respected and valued by other team members, and having those employees who feel positive about their racial or ethnic identity in the organization (Lindsay, Rezai, Shen, & Lyons, 2020; Sakdiyakorn & Wattanacharoensil, 2018).

In contrast to social identity theory, the similarity attraction theory is one of the oldest perspectives that is applied in relational demography research and have application to organizational performance. This theory was formed when a parallel connections were found between individuals’ social status, demographic factors, beliefs and values (John F. et al., 2017). The similarity attraction theory suggests that parallel personal traits between individuals relates directly to their interpersonal attraction and creates positive expectations, which eventually results in higher collaboration and admiration for each other (Mun & Jung, 2018; Oliveira & Cabral-Cardoso, 2017). This attraction grows as more similar attributes are discovered, on the contrary, repulsion arises with the increase in dissimilarities. Therefore, individuals who share highly similar attributes would have more attraction to each other that enhances organizational performance due to leisure enjoyments and positive working relations between individuals (John F. et al., 2017; Mun & Jung, 2018). Moreover, Optimal distinctiveness theory also discuss the differences and similarities among in-groups and out-groups and strives for social identification and optimal distinctiveness (Bizri, 2018; Hsiao, Ma, Lloyd, & Reid, 2019).

Moreover, within the organizations the similarities among employees have been connected to different positive effects i.e. improved communication, reduced employee turnover intentions, and improved team work (P.A, 2018; Ng & Sears, 2020). Conversely, the dissimilarities have been associated with potential negative effects that hinders the organizational performance due to feeble peer relations and social integration (Páez-Avilés, Rijnsoever, Juanola-Feliu, & Samitier, 2018). Former researches add further evidence that due to more similarity in a dyad relationship between managers and employees may enhance the organizational performance (Urbancová, Hudáková, & Fajčíková, 2020). For instance, (Tsui et al., 1992) established a positive correlation between similarity attractions, interpersonal attractions, role effectiveness, and the performance evaluations of those having similar demographics i.e. ethnicity, educational background, gender, age etc.

The availability of the contrasting theories and research findings make diversity more interesting for research investigation. These findings also proclaim that diversity and its effects have remained different for different settings and may results differently, due to social, cultural, psychological and emotional reasons. Therefore, situational analysis may be needed for managing diversity to get fruitful results.

**Research Hypothesis**

Due to growing pressures in corporate world accompanied with fierce competition, new technology and globalization, organizations tend to find ways that ensures them sustainable competitive edge over others. As global focus and communication is increasing, one way to have a competitive edge is through embracing diversity at workplace. The diverse workforce should have ability to effectively deal with as well as adapt to the ever changing needs of stakeholders. According to prior studies, diversity at workplace has positive impact on Organizational performance that is mainly the result of greater innovations, better organizational flexibility, rational decision making and problem solving, eventually results in improved organizational performance (Richard S. Allen et. al, 2007). Even different research findings recommend that diversity bring beauty to organization and increase Organizational performance. Age, gender, educational and ethnic diversity adds to the synergetic pragmatism of the projects and organization (Barros, 2019; AndreSøraa, et al., 2020). These finding leads us to the stance that

**H1: Diversity has positive significant impact on project performance.**

Diversity is considered as a beauty of the universe. Individuals with unique capabilities and capacities bring values, tacit and indigenous knowledge to the organization, which synergies, complement one another, which improve organizational performance. 21^st^ century arena is the competitive landscape where sustainability and organizational growth depends upon the diverse abilities and skills of a leader and workers, for getting expected results, better image, and above average returns. Previous results finding maintain that if leadership effective diversity management at workplace, it adds to both organizational and Organizational performance (AndreSøraa, et al., 2020; Bizri, 2018). Diversity, which has become an integral part of every organization and project in this unified world, needs better leadership expertise to get it managed at micro and macro level (Hsiao, Ma, Lloyd, & Reid, 2019; Manoharan & Singal, 2017). The former researches supports that leader’s expertise i.e. leading employees with respect regardless of their caste and creed, leading them with self-assurance, shaping their behavior is a positive way results in enhanced employee performance, which eventually reflects in increased Organizational performance (Szu-Fang. C, 2013). The findings lead us to

**H2: Diversity has positive impact on organizational performance with leadership expertise.**

Leadership is expected to give new directions, new philosophies, optimism and enthusiasm to the knowledge workers. They shape and reshape not only knowledge workers, but also organizational routines and flows, which bring persuasion, motivation to the workers, who put their head and heart in getting things done (Carroll, 2012). Moreover, the relationship between leadership expertise and Organizational performance is strengthened through convincing diversity beliefs. The diverse beliefs accompanied with respect and openness towards acceptance for everyone in the organizational could result in more satisfied, more committed, better employee performance and hence Organizational performance (Patrick, H., & Kumar, V., 2012). A team with diverse beliefs require those leaders who can guide the entire team in a productive manner towards same direction. However, prior researches suggests that recognizing beliefs, values, strengths and weaknesses of team members is one of the essential traits of leader that is required to delegate responsibilities appropriately to each member. This could be managed through smooth and open communication that eventually enhance the overall performance of organization (Wee, J., & Morse, O.07, 2007). This findings employees that

**H3: Diversity beliefs moderate’s relationship between leadership expertise and organizational performance.**

Furthermore, in today’s world, organizations are bringing people from different cultures in order to boost the creativity, stock of knowledge and rational problem-solving approaches. Consequently, the leaders in this 21^st^ century has become highly alarmed with diversity management in the organizations (Yukl, G., 2001). It is believed that diversity at workplace has positive impact on the organizational performance, and this relationship is mediated by the leadership expertise. According to prior researches, the organizational leaders play vital role in forming and promotion the workplace culture, free of prejudice and personal biases. The workforce mainly follows leaders to set the perspective wherein they would work in an organizational setting, thus, forming such an environment that imitates respect, ethical behavior, understanding and encouraging cross-cultural values leads to better organizational performance. However, this relationship is moderated by the diversity beliefs. Everyone in the organization do not hold same values and beliefs, but a true leader who can determine varied beliefs of employees and manage diversity in way that is convincing for each team member can help organizations towards reaching new heights (Aggarwa, R., 2011). The research findings lead to the hypothesis that

H4: Diversity has significant impact on Organizational performance with mediation of leadership expertise and moderation of diversity beliefs.

**2.5 Conceptual framework**

Diversity

Age Diversity

Gender Diversity

Educational diversity

Ethnic Diversity

Organizational Performance

Diversity Beliefs

Leadership Expertise

Figure 1: Conceptual Model

**Research Methodology**

The main objective of this research is to assess the impact of workforce diversity on Organizational performance in the presence of leadership expertise as mediating variable and diversity beliefs as moderating variable. Keeping in concerns the objectives of study, the quantitative approach was applied to get research questions’ answer. In the first phase, the comprehensive systematic literature review was conducted to explore all the dimensions of diversity, its role towards Organizational performance. Moreover, diversity dimensions were studied with reference to leadership expertise and diversity beliefs in order to evaluate the mediation and moderation effect. In the second phase, questionnaire was developed and cross-sectional approach was used for data collection. The unit of analysis was individual representing individual. Data was collected from employees employed in services sector and projectized organizations based in major cities of Pakistan (Islamabad, Lahore and Karachi). Data was collected from organizations working in educational Sector, Hospitality Sector and Banking Sector. Convenient sampling was used for data collection. Consequently, preliminary analysis will be done through SPSS software and Smart PLS was used for the data analysis. Total of 250 questionnaires were distributed among the respondents and 205 were get backed. Questionnaire were scanned for data completion and missing values. 17 questionnaires were discarded due having more than 10% to missing values. Similarly, 12 more questionnaires were sidelined due to outliers and the rest were analyzed for results generations.

**RESULTS AND FINDINGS**

**Demographics of the Study**

Table 2 contains demographic details of the respondents. Among respondents 97 were male and 79 were female. Most of the respondents were among 30-35, means were having active field engagement of organizational projects and operations. similarly, they were having quite good experience of organization and effective learning. Moreover, the data reliability can also be promised that all the respondents were having reasonable educational background, beside organizational knowledge and experiences.

Table 2

Demographics of the Respondents

| **Gender** | **Frequency** | **Percent** |
| --- | --- | --- |
| Male | 97 | 55.114 |
| Female | 79 | 44.886 |
| **Age** | **Frequency** | **Percent** |
| 30-35 | 114 | 64.773 |
| 36-40 | 40 | 22.700 |
| 41-above | 22 | 12.500 |
| **Service** | **Frequency** | **Percent** |
| 1-5 years | 82 | 46.591 |
| 5-10 years | 60 | 34.100 |
| Other | 34 | 19.318 |
| **Qualifications** | **Frequency** | **Percent** |
| Bachelor | 44 | 25.000 |
| Master | 68 | 38.636 |
| Ph.D. | 32 | 18.182 |
| Other | 32 | 18.182 |

**Instrument validity**

Table 3 indicates loading of the factors, and all the items are following in the acceptable range, i.e. greater than 0.70. Similarly, Average Variance Extracted (AVE) are falling 0.612-0.678 for the constructs, which is highly reliable magnitude. Moreover, the values of Composite Reliability (CR) ranges are from 0.862 till 0.947, which is highly consistent, which satisfy the criteria of convergent validity. Furthermore, predictive accuracy, effect size and predictive relevance were conducted for goodness of fit, and their values found falling in acceptable range.

Table 3

Confirmatory Factor Analysis for Research Constructs

| Constructs | No. of item* | Factor Loading** | AVE | CR |  | Goodness of Fit Indices | | |
| --- | --- | --- | --- | --- | --- | --- | --- | --- |
|  |  |  |  |  | χ^2^/df | Q^2^ | R^2^ | F^2^ |
| OL | 3 | .738 - .911 | .678 | .911 | 1.171 | 0.244 | 0.452 | 0.294  0.202  0.083  0.381  0.021  0.201  0.217 |
| AD | 4 | .754 - .926 | .671 | .932 | 2.692 | 0.171 | 0.437 |  |
| ED | 5 | .833 - .855 | .753 | .927 | 1.273 | 0.204 | 0.445 |  |
| GD | 4 | .837- .840 | .764 | .947 | 1.114 | 0.365 | .476 |  |
| EDD | 7 | .826 - .839 | .784 | .937 | 2.925 | 0.272 | 0.229 |  |
| LE | 4 | .744 - .840 | .612 | .862 | 1.817 | 0.293 | 0.427 |  |
| DB | 3 | .714 - .869 | .674 | .884 | 2.903 | 0.213 | 0.341 |  |

#### **Discriminant Validity:** **Fornell-Larcker Criterion**

Discriminant validity of the constructs was checked using Fornell-Larcker Criterion***.*** Discriminant validity is considered confirm, if correlation among constructs does not exceed 0.85 and also, the square root of AVEs is greater than the correlation of other constructs. Table 4 maintains all values, which are less than 0.85 and their square root of AVEs are greater than off-diagonals values of their constructs. These details provide the satisfaction of the discriminant validity.

Table 4

Square roots of AVEs

| **Constructs** | **AD** | **DB** | **ED** | **EDD** | **GD** | **LE** | **OP** |
| --- | --- | --- | --- | --- | --- | --- | --- |
| AD | 0.759 |  |  |  |  |  |  |
| DB | 0.593 | 0.844 |  |  |  |  |  |
| ED | 0.650 | 0.543 | 0.777 |  |  |  |  |
| EDD | 0.596 | 0.591 | 0.571 | 0.758 |  |  |  |
| GD | 0.638 | 0.412 | 0.448 | 0.575 | 0.820 |  |  |
| LE | 0.730 | 0.690 | 0.653 | 0.623 | 0.564 | 0.837 |  |
| OP | 0.726 | 0.706 | 0.608 | 0.602 | 0.546 | 0.833 | 0.847 |

**Discriminant Validity: HTMT Criterion**

HTMT refers to the average of the correlations of indicators between different constructs, relative to the average of the correlations of indicators within the same construct (Ashkanasy, 2016; Canessa-Terrazas and Morales-Flores, 2017). It measures the discriminant validity between construct of the instrument. Its cut-off values are (1). the conservative ratio of 0.9 (HTMT.90) advocated by Gold et al. (2001), or (2). the stringent ratio of 0.85 (HTMT.85) advocated by Kline (2011). The cut-off ratio of 0.85 (HTMT.85) is however noted as offering the best criterion when compared to all other methods of assessing discriminant validity (Ramayah et al., 2016). Thus, any inter-construct ratio greater than 0.85 would be considered as having poor discriminant validity. The HTMT ratios obtained in this study as shown in Table 4 clearly indicates that there is no problem of discriminant validity between the constructs. Table 4 indicates that all the values of the HTMT are lower than their both cut-off values.

Table 5

HTMT Values

| **Constructs** | **AD** | **DB** | **ED** | **EDD** | **GD** | **LE** | **OP** |
| --- | --- | --- | --- | --- | --- | --- | --- |
| AD |  |  |  |  |  |  |  |
| DB | 0.762 |  |  |  |  |  |  |
| ED | 0.792 | 0.634 |  |  |  |  |  |
| EDD | 0.759 | 0.723 | 0.671 |  |  |  |  |
| GD | 0.806 | 0.504 | 0.512 | 0.701 |  |  |  |
| LE | 0.834 | 0.834 | 0.739 | 0.734 | 0.657 |  |  |
| OP | 0.811 | 0.809 | 0.710 | 0.734 | 0.658 | 0.789 |  |

Figure 2: PLS-Algorithm result with outer loadings and AVE

**Hypothesis Testing**

The path estimation or hypothetical relations was performed to observe the significance relationship in the inner path model. The entire hypothetical path in the framework was examined through the regression coefficient (β). By using PLS Bootstrap technique the value of β was checked to observe the proposed hypotheses in the structural model. Table 5 presented the path coefficient assessment result where out of 10 direct hypotheses 6 hypotheses were supported and 4 hypotheses were not supported. The supported hypotheses are significant at least at the level of 0.05, have expected sign directions (i.e., positive) and consist of a path coefficient value (β) ranging from 0.181 to 0.515. In Table 6 displayed that all 6 direct relationships were significant as the p value is less than 0.05 and the t-value is higher than 1.96 which also depicted in figure 2. However, the other 4 hypotheses were unsupported due to the p-value were higher than 0.05 and t-value were less than 1.96.

In the case of moderating hypothesis, DB does not moderate the relationship between LE and OP. Therefore, it confirms that DB doesn’t play any significant moderating role on the relationship between LE and OP.

Table 6

Direct and moderating Hypothesis

| **Hypotheses** | **OS** | **SM** | **SD** | **T** | **P Values** | **Decision** |
| --- | --- | --- | --- | --- | --- | --- |
| AD -> LE | 0.401 | 0.396 | 0.082 | 4.865 | 0.000 | Significant |
| AD -> OP | 0.181 | 0.187 | 0.091 | 1.992 | 0.047 | Significant |
| DB -> OP | 0.212 | 0.217 | 0.079 | 2.674 | 0.008 | Significant |
| ED -> LE | 0.242 | 0.247 | 0.077 | 3.165 | 0.002 | Significant |
| ED -> OP | 0.013 | 0.013 | 0.087 | 0.151 | 0.880 | Not Significant |
| EDD -> LE | 0.195 | 0.191 | 0.078 | 2.521 | 0.012 | Significant |
| EDD -> OP | 0.021 | 0.017 | 0.062 | 0.331 | 0.741 | Not Significant |
| GD -> LE | 0.087 | 0.096 | 0.089 | 0.976 | 0.330 | Not Significant |
| GD -> OP | 0.037 | 0.039 | 0.066 | 0.568 | 0.570 | Not Significant |
| LE -> OP | 0.515 | 0.508 | 0.087 | 5.952 | 0.000 | Significant |
| LE*DB -> OP | -0.005 | -0.005 | 0.027 | 0.186 | 0.853 | Not Significant |

**Mediation Hypothesis**

For the mediating analysis, the bootstrapping technique was applied for this research which was suggested by Hair et al. (2013). The results of the mediation analysis are presented in Table 7 where among the four mediating hypotheses three mediating hypotheses were supported and one was not supported. The mediating path AD -> LE -> OP, ED -> LE -> OP, and EDD -> LE -> OP were significant as the p-value was less than 0.05 and the values of LL and UL doesn’t have zero (0) in between which confirms mediating effect. However, the other mediating path GD -> LE -> OP is not significant as the p value is higher than 0.05 and the zero (0) exist in between LL and UL. In addition, among the three hypotheses the AD -> LE -> OP path was showing partially mediated as the direct hypothesis was significant, however the other two significant path were full mediated as their direct relationships were not significant.

Table 7

Mediation Hypothesis

| **Hypothesis** | **OS (Beta)** | **95% Confidence  Interval** | | **T** | **P Values** | **Decision** | **Mediation** |
| --- | --- | --- | --- | --- | --- | --- | --- |
|  |  | **LL** | **UL** |  |  |  |  |
| AD -> LE -> OP | 0.206 | 0.122 | 0.345 | 3.743 | 0.000 | Significant | Partial Mediation |
| ED -> LE -> OP | 0.125 | 0.055 | 0.222 | 2.964 | 0.003 | Significant | Full Mediation |
| EDD -> LE -> OP | 0.101 | 0.025 | 0.210 | 2.226 | 0.026 | Significant | Full Mediation |
| GD -> LE -> OP | 0.045 | -0.034 | 0.150 | 0.973 | 0.331 | Not Significant | No Mediation |

Figure 3: Bootstrapping result with inner t-values

**Conclusion and Future Recommendations**

After many years of talking about workplace diversity still there is considerable misperception over what actually diversity. It consists of the broad definitions of diversity seek inclusion, but don’t allow to identify the difference between social and functional diversity. However, the narrow definitions may be used to explain either of these, but exclude a lot of diverse considerations. These differences mainly let organizations to face confusion, thus, it is of utmost importance for organizations to differentiate between social and functional diversity, and treat these forms differently.

The present research provides wide understanding on the prevailing diversity scenario in the service sector of Pakistan. The research indicates that the diversity of three demographic variables i.e. ethnicity, gender and educational background doesn’t impact significantly on the organizational performance, while only age diversity has significant impact on the organizational performance. Therefore, keeping in mind gender, ethnicity and educational background it is concluded that employees neither admire the diversity at workplace nor do they criticize it. The same can be indicated by the moderating hypothesis, that diversity beliefs plays no significant role in improving organizational performance. This study challenges the finding of previous studies given in the literature review sections, which proclaims that diversity and diversity beliefs have significant effect on organizational performance, therefore, beside multi-national companies, in today’s business arena small, medium and regional (local) organizations prefer to engage work force with diverse social, cultural and ethnic background, bring multi-facet experiences, learning, tacit and explicit knowledge to the organizational, to boom effectiveness and efficiencies, to face challenges and accept future challenges. This may be due to regional and cultural factors, that diversity beliefs are not promoting organizational performance, which may be explored in future. Moreover, this study indicates that leadership expertise play significant mediating role and diversity beliefs play significant moderating role in organizational performance.

The workplace diversity is becoming one of the most popular ways to evaluate the organizational performance. Thus, conducting training and creating the awareness regarding diversity will lead to more productivity. Managing the diversity at workplace considers leveraging and respecting the cultural differences in employee’s competencies, ideas, and innovativeness in order to persuade them to contribute towards common goal, and doing it in a way that gives competitive edge to organizations. Hence, it is recommended to encourage more of diversified workforce and create awareness to increase the organizational performance. In addition, this research has focused on diversity beliefs as a moderating variable, however, the future researches can also be conducted on organizational justice as a moderator between diversity dimensions and organizational performance. Moreover, in current paper the social traits of diversity have been studied, providing opportunity or gap to study functional diversity traits in future as well.

**Ethical Consent**

The study was approved from the ethical committee of SZABIST Larkana Campus. The consent was informed and the information were collected though approved structured questionnaire. Moreover, there is no and anykind of disagreement among the authors.

# **References**

Albert, M., Balve, P., & Spang, K. (2017). Evaluation of Organizational performance : a structured literature review. *International Journal of Managing Projects in Business, Vol. 10 No. 4, https://doi.org/10.1108/IJMPB-01-2017-0004*, 796-821.

Ali, M., & M.Konrad, A. (2017). Antecedents and consequences of diversity and equality management systems: The importance of gender diversity in the TMT and lower to middle management. *European Management Journal Volume 35, Issue 4*, 440-453.

gtbetu6ytrAnaGarcía-Granero, Fernández-Mesa, A., Jansen, J. J., & Vega-Jurado, J. (2018). Top management team diversity and ambidexterity: The contingent role of shared responsibility and CEO cognitive trust. *Long Range Planning Volume 51, Issue 6*, 881-893.

Barros, A. R. (2019). *The link between social responsibility and diversity in the workplace.* Canada : ISCTE Business School.

Bizri, R. (2018). Diversity management and OCB: the connection evidence from the Lebanese banking sector. *Equality, Diversity and Inclusion, Vol. 37 No. 3, https://doi.org/10.1108/EDI-03-2017-0059*, 233-253.

Boamah, S. (2018). Linking Nurses' Clinical Leadership to Patient Care Quality: The Role of Transformational Leadership and Workplace Empowerment. *Canadian Journal of Nursing Research, Vol 50, Issue 1*, 219-234.

Butticè, V., Colombo, M. G., & Wright, M. (2017). Serial Crowdfunding, Social Capital, and Organizational performance . *Entrepreneurship Theory and Practice, Vol 41, Issue 2*, 183-207.

Campbell Leaper. (2011). More Similarities than Differences in contemporary Theories of social development? *Advances in Child Development and Behavior*.

Dahanayake, P., Rajendran, D., Selvarajah, C., & Ballantyne, G. (2018). Justice and fairness in the workplace: a trajectory for managing diversity. *Equality, Diversity and Inclusion: An International Journal Vol. 37 No. 5*, 470-490.

Duchek, S., Raetze, S., & Scheuch, I. (2020). The role of diversity in organizational resilience: a theoretical framework. *Business Research Isuue 13*, 387–423.

Georgakakis, D., Greve, P., & Ruigrok, W. (2017). Top management team faultlines and firm performance: Examining the CEO-TMT interface. *The Leadership Quarterly Volume 28, Issue 6*, 741-758.

Gomez, L., & Bernet, P. (2019). Diversity improves performance and outcomes. *Journal of the National Medical Association Volume 111, Issue 4*, 383-392.

Heavey, C., & Simsek, Z. (2017). Distributed Cognition in Top Management Teams and Organizational Ambidexterity: The Influence of Transactive Memory Systems. *Journal of Management, Vol 43, Issue 3*, 1375-1382.

Hennekam, S., Bacouel-Jentjens, S., & Yang, I. (2019). Ethnic diversity management in France: a multilevel perspective. *International Journal of Manpower, Vol. 40 No. 1, https://doi.org/10.1108/IJM-10-2017-0272*, 120-134.

Hogg, M. A. (2001). A Social Identity Theory of Leadership. *Personality and Social Psychology Review, vol 5, Issue 3*, 253-271.

Hogg, M. A. (2011). Subjective Uncertainty Reduction through Self-categorization: A Motivational Theory of Social Identity Processes. *European Review of Social Psychology Volume 11, 2000 - Issue 1*, 223-255.

Hogg, M. A., & Terry, D. I. (2000). Social Identity and Self-Categorization Processes in Organizational Contexts. *Academy of Management ReviewVol. 25, No. 1*, 234-254.

Hornsey, M. J. (2008). Social Identity Theory and Self‐categorization Theory: A Historical Review. *Social and Personality Psychology Compass 2/1*, 204–222.

Hsiao, A., Ma, E. (., Lloyd, K., & Reid, S. (2019). Organizational Ethnic Diversity’s Influence on Hotel Employees’ Satisfaction, Commitment, and Turnover Intention: Gender’s Moderating Role. *Journal of Hospitality & Tourism Research, Vol 44, Issue 1*, 1-22.

Jankelová, N., Joniaková, Z., Procházková, K., & Blštáková, J. (2020). Diversity Management as a Tool for Sustainable Development of Health Care Facilities. *Sustainability 2020, 12(13), https://doi.org/10.3390/su12135226*, 1-22.

Jansson, F., & Bursell, M. (2018). Social consensus influences ethnic diversity preferences. *Social Influence Volume 13,Issue 4*, 192-208.

Jeong, S. (2020). A cross‐level analysis of organizational knowledge creation: How do transformational leaders interact with their subordinates' expertise and interpersonal relationships? *Human Resource Development Quarterly*, 1-20.

Jin, M., Lee, J., & Lee, M. (2017). Does leadership matter in diversity management? Assessing the relative impact of diversity policy and inclusive leadership in the public sector. *Leadership & Organization Development Journal, Vol. 38 No. 2, https://doi.org/10.1108/LODJ-07-2015-0151*, 303-319.

John F. et al. (2017). Social identities and social context: social attitudes and personal well-being . *Emerald Insight* .

Joubert, Y. T. (2017). Workplace diversity in South Africa: Its qualities and management. *Journal of Psychology in Africa Volume 27, Issue 4*, 367-371.

Kalargyrou, V., & Costen, W. (2017). Diversity management research in hospitality and tourism: past, present and future. *International Journal of Contemporary Hospitality Management, Vol. 29 No. 1. https://doi.org/10.1108/IJCHM-09-2015-0470*, 68-114.

Kan, S. K., & Kaplan, S. (2019). Working toward gender diversity and inclusion in medicine: myths and solutions. *The Lancet Volume 393, Issue 10171, 9–15* , 579-586.

Kato, T., & Kodama, N. (2018 ). The Effect of Corporate Social Responsibility on Gender Diversity in the Workplace: Econometric Evidence from Japan. *BJIR Symposium on Corporate Social Responsibility and Labour Standards, Volume56, Issue1*, 99-127.

Khan, M. S., Lakha, F., Tan, M. M., Singh, S. R., Quek, R. Y., Han, E., . . . Legido-Quigley, H. (2019). More talk than action: gender and ethnic diversity in leading public health universities. *The Lancet Volume 393, Issue 10171, 9–15*, 594-600.

Krivkovich, A., Nadeau, M.-C., Robinson, K., Robinson, N., Starikova, I., & Yee, L. (2018). *Women in the Workplace 2018.* London : McKinsey.

Kundu, S. C., & Mor, A. (2017). Workforce diversity and organizational performance: a study of IT industry in India. *Employee Relations, Vol. 39 No. 2, https://doi.org/10.1108/ER-06-2015-0114*, 160-183.

Laurence, J., Schmid, K., & Hewstone, M. (2018). Ethnic Diversity, Inter-group Attitudes and Countervailing Pathways of Positive and Negative Inter-group Contact: An Analysis Across Workplaces and Neighbourhoods. *Social Indicators Research volume 136*, 719–749.

Lindsay, S., Rezai, M., Shen, W., & Lyons, B. (2020). A disability disclosure simulation as an educational tool. *Equality, Diversity and Inclusion, Vol. 39 No. 8, https://doi.org/10.1108/EDI-12-2019-0292*, 865-879 .

Luanglath, N., Ali, M., & Mohannak, K. (2019). Top management team gender diversity and productivity: the role of board gender diversity. *Equality, Diversity and Inclusion, Vol. 38 No. 1, https://doi.org/10.1108/EDI-04-2018-0067*, 71-86.

M.Carvalho, M., & Jr, R. R. (2017). Can project sustainability management impact Organizational performance ? An empirical study applying a contingent approach. *International Journal of Project Management Volume 35, Issue 6*, 1120-1132.

Madsen, S. R., Townsend, A., & Scribner, R. T. (2020). Strategies That Male Allies Use to Advance Women in the Workplace. *The Journal of Men’s Studies, Vol 28, Issue 3*, 145-162.

Makudza, F., Muchongwe, N., & Dangaiso, P. (2020). Workforce Diversity: A Springboard for Employee Productivity and Customer Experience. *Journal of Industrial Disribution & Business Vol 11 No 10* , 49-58.

Malik, A. R., & Singh, P. (2017). Transformational leadership and cultural minorities: a conceptual model. *European Business Review, Vol. 29 No. 5, https://doi.org/10.1108/EBR-12-2015-0181*, 500-514.

Manoharan, A., & Singal, M. (2017). A systematic literature review of research on diversity and diversity management in the hospitality literature. *International Journal of Hospitality Management Volume 66*, 77-91.

Martinez, M. G., Zouaghi, F., & Marco, T. G. (2017). Diversity is strategy: the effect of R&D team diversity on innovative performance. *Effects of individuals and teams on R&D outcomes, Volume 47, Issue 2* , 311-329.

Mousa, M., Massoud, H. K., & Ayoubi, R. M. (2020). Gender, diversity management perceptions, workplace happiness and organisational citizenship behaviour. *Employee Relations, Vol. 42 No. 6, https://doi.org/10.1108/ER-10-2019-0385*, 1249-1269.

Mun, E., & Jung, J. (2018). Change above the Glass Ceiling: Corporate Social Responsibility and Gender Diversity in Japanese Firms. *Administrative Science Quarterly, Vol 63, Issue 2*, 409-440.

Ng, E. S., & Sears, G. J. (2020). Walking the Talk on Diversity: CEO Beliefs, Moral Values, and the Implementation of Workplace Diversity Practices. *Journal of Business Ethics volume 164*, 437–450.

Nuscheler, D., Engelen, A., & Zahra, S. A. (2019). The role of top management teams in transforming technology-based new ventures' product introductions into growth. *Journal of Business Venturing Volume 34, Issue 1*, 122-140.

Oliveira, E., & Cabral-Cardoso, C. (2017). Older workers’ representation and age-based stereotype threats in the workplace. *Journal of Managerial Psychology, Vol. 32 No. 3, https://doi.org/10.1108/JMP-03-2016-0085*, 254-268.

Ou, A. Y., Seo, J., Choi, D., & Hom, P. W. (2016). When Can Humble Top Executives Retain Middle Managers? The Moderating Role of Top Management Team Faultlines. *Academy of Management JournalVol. 60, No. 5*, 213-238.

P.A, G. (2018). Age Diversity in the Workplace. *Diversity and Inclusion in the Global Workplace* , 31-47.

Páez-Avilés, C., Rijnsoever, F. J., Juanola-Feliu, E., & Samitier, J. (2018). Multi-disciplinarity breeds diversity: the influence of innovation project characteristics on diversity creation in nanotechnology. *The Journal of Technology Transfer volume 43*, 458–481.

Park, J. S.-Y. (2020). Translating Culture in the Global Workplace: Language, Communication, and Diversity Management. *Applied Linguistics, Volume 41, Issue 1*, 109-128.

PráMartensa, C. D., Machado, F. J., Martens, M. L., Silva, F. Q., & Freitas, H. M. (2018). Linking entrepreneurial orientation to Organizational performance . *International Journal of Project Management Volume 36, Issue 2*, 255-266.

Profili, S., Innocenti, L., & Sammarra, A. (2017). A Conceptual Framework of Age Diversity Climate. *Age Diversity in the Workplace (Advanced Series in Management, Vol. 17), https://doi.org/10.1108/S1877-636120170000017006*, 95-116.

PrósperoSanchez, O., Terlizzi, M. A., & Moraes, H. R. (2017). Cost and time project management success factors for information systems development projects. *International Journal of Project Management Volume 35, Issue 8*, 1608-1626.

Radujković, M., & Sjekavica, M. (2017). Project Management Success Factors. *Procedia Engineering Volume 196*, 607-615.

Roberson, Q. M. (2019). Diversity in the Workplace: A Review, Synthesis, and Future Research Agenda. *Annual Review of Organizational Psychology and Organizational Behavior Vol. 6:69-88*, 69-88.

Sakdiyakorn, M., & Wattanacharoensil, W. (2018). Generational Diversity in the Workplace: A Systematic Review in the Hospitality Context. *Cornell Hospitality Quarterly vol 59, Issue 2*, 393=412.

Seo, G., Huang, W., & Han, S.-H. C. (2017). Conceptual Review of Underrepresentation of Women in Senior Leadership Positions From a Perspective of Gendered Social Status in the Workplace: Implication for HRD Research and Practice. *Human Resource Development Review, vol 16, Issue 1*, 35-59.

Seong, J. Y., & Hong, D.‐S. (2018). Age diversity, group organisational citizenship behaviour, and group performance: Exploring the moderating role of charismatic leadership and participation in decision‐making. *Human Resource Management Journal, Volume28, Issue 4*, 621-640.

Sinnema, C., Daly, A. J., Liou, Y.-H., & Rodway, J. (2020). Exploring the communities of learning policy in New Zealand using social network analysis: A case study of leadership, expertise, and networks. *International Journal of Educational Research Volume 99*, 22-46.

Tajfel & Turner. (1985). The social identity theory of intergroup behavior. *Psychology of intergroup relations*, 7-24.

Tamunomiebi, M. D., & John-Eke, E. C. (2020). Workplace Diversity: Emerging Issues in Contemporary Reviews. *International Journal of Academic Research in Business and Social Sciences Vol. 1 0 , No. 2*, 255 - 265.

Toscano, F., Giusino, D., & Pordanjani, T. R. (2002). Revisiting the Women Workplace Culture Scale: Validation and Psychometric Properties of a Three-Factor Structure in an Iranian Study Sample. *European journal of investigation in health psychology and education, 10(3)*, 915-934.

Trepte, S., & Loy, L. S. (2017). Social Identity Theory and Self‐Categorization Theory. *The International Encyclopedia of Media Effects*, 20-43.

Tsui et al. (1992). Being different: Relational demography and organizational attachment. *Administrative Science Quarterly*, 549–579.

Urbancová, H., Hudáková, M., & Fajčíková, A. (2020). Diversity Management as a Tool of Sustainability of Competitive Advantage. *Sustainability, 12(12), https://doi.org/10.3390/su12125020*, 24-39.

Veronica, V., Alamsjah, F., Elidjen, E., & Kosasih, W. (2020). Organizational Culture And Technological Change Impact On Organizational Innovativeness With A Moderating Effect Of Age Diversity. *advances in transportation and logistics research*, 844-855.

Wang, M., & Fang, Y. (2020). Age Diversity in the Workplace: Facilitating Opportunities With Organizational Practices. *Public Policy & Aging Report, Volume 30, Issue 3*, 119–123.

Wang, X., & Dass, M. (2017). Building innovation capability: The role of top management innovativeness and relative-exploration orientation. *Journal of Business Research Volume 76*, 127-135.

Wua, G., Liu, C., Zhao, X., & Zuo, J. (2017). Investigating the relationship between communication-conflict interaction and Organizational performance among construction project teams. *International Journal of Project Management Volume 35, Issue 8*, 1466-1482.

Zahid, M., Rahman, H. U., Ali, W., Khan, M., Alharthi, M., Qureshi, M. I., & Jan, A. (2020). Boardroom gender diversity: Implications for corporate sustainability disclosures in Malaysia. *Journal of Cleaner Production Volume 244*, 234-249.

Zhao, J. L., Chen, S., Jiang, W., & Shi, T. L. (2017). Gender Diversity on Boards and Firms’ Environmental Policy. *business strategy and the environment, Volume26, Issue3* , 306-315.

ZhenShao, Feng, Y., & Hu, Q. (2017). Impact of top management leadership styles on ERP assimilation and the role of organizational learning. *Information & Management Volume 54, Issue 7*, 902-919.
