# Supplementary material for: Diversity impact on organizational performance: Moderating and mediating role of diversity beliefs and leadership expertise
Source: PLoS One. 2022 Jul 25;17(7):e0270813. doi: 10.1371/journal.pone.0270813 (PMC9578724; doi:10.1371/journal.pone.0270813)
Supplement: Attachment — Submitted filename: reviewers comments and responses.docx [file pone.0270813.s003.docx]

| s. no | Reviewers’ suggestions | Responses to reviewers’ comments | Page no |
| --- | --- | --- | --- |
| 1 | The manuscript must describe a technically sound piece of scientific research with data that supports the conclusions. Experiments must have been conducted rigorously, with appropriate controls, replication, and sample sizes. The conclusions must be drawn appropriately based on the data presented. Partly | **Suggestion accepted and incorporated**  Thanks for the comments and appreciation.  The paper was revisited and all the analyses were done again, the section was improved, keeping in mind the objectives and research question. | Pg 5-9 |
| 2. | Has the statistical analysis been performed appropriately and rigorously? Yes | **Suggestion accepted and incorporated**  Thanks for the comments and appreciation.  The paper was revisited and all the analyses were done again, the section was improved, keeping in mind the objectives and research question. | Pg 5-9 |
| 3 | Is the manuscript presented in an intelligible fashion and written in standard English?  PLOS ONE does not copyedit accepted manuscripts, so the language in submitted articles must be clear, correct, and unambiguous. Any typographical or grammatical errors should be corrected at revision, so please note any specific errors here.  Reviewer #1: No | **Suggestion accepted and incorporated**  The whole paper was revisited and the help of the expert/native speaker was also incorporated. Additionally, language software was also used for language and grammar improvement. | Whole paper |
| 4. | Reviewer #1: The study is interesting, and the results are useful for diversity related research. However, the manuscript needs to be thoroughly checked for language including grammar. Detailed review comments are attached. | **Suggestion accepted and incorporated**  Thanks for your appreciation, the whole paper was revisited and the suggestions were incorporated | Whole paper |
| 5 | Reviewer #2: Dear Authors You get a minor revision, make a revision immediately, pay attention to the marked reviews, the introduction is quite clear but there are some things that need to be improved, the methods, data processing and others are quite good Thank you | **Suggestion accepted and incorporated**  Thanks for your appreciation, the whole paper was revisited and the suggestions were incorporated | Whole paper |
